# Novel Coronavirus: Current Understanding of Clinical Features, Diagnosis, Pathogenesis, and Treatment Options

**DOI:** 10.3390/pathogens9040297

**Published:** 2020-04-17

**Authors:** Mohammad Ridwane Mungroo, Naveed Ahmed Khan, Ruqaiyyah Siddiqui

**Affiliations:** Department of Biology, Chemistry and Environmental Sciences, College of Arts and Sciences, American University of Sharjah, University City, Sharjah 26666, UAE; ridwanemungroo@gmail.com (M.R.M.); rsiddiqui@aus.edu (R.S.)

**Keywords:** coronavirus, COVID-19, SARS-CoV-2, 2019-nCoV, treatment, diagnosis, transmission

## Abstract

Since December 2019, coronavirus disease 2019 (COVID-19) caused by severe acute respiratory syndrome coronavirus 2 (SARS-CoV-2) has resulted in devastating consequences worldwide and infected more than 350,000 individuals and killed more than 16,000 people. SARS-CoV-2 is the seventh member of the coronavirus family to affect humans. Symptoms of COVID-19 include fever (88%), cough (68%), vomiting (5%) and diarrhoea (3.7%), and transmission of SARS-CoV-2 is thought to occur from human to human via respiratory secretions released by the infected individuals when coughing and sneezing. COVID-19 can be detected through computed tomography scans and confirmed through molecular diagnostics tools such as polymerase chain reaction. Currently, there are no effective treatments against SARS-CoV-2, hence antiviral drugs have been used to reduce the development of respiratory complications by reducing viral load. The purpose of this review is to provide a comprehensive update on the pathogenesis, clinical aspects, diagnosis, challenges and treatment of SARS-CoV-2 infections.

## 1. Introduction

Since December 2019, an outbreak caused by a pathogen resulting in a respiratory disease occurred in Wuhan, China. The disease was termed coronavirus disease 2019 (COVID-19) by the World Health Organization (WHO) and the causative agent was identified as severe acute respiratory syndrome coronavirus 2 (SARS-CoV-2) [1]. The disease is now a global threat; and on 11 March 2020, WHO recognized the outbreak as a pandemic [2] and SARS-CoV-2 has infected more than 372,757 and killed 16,231 people according to WHO [2]. 

Coronaviruses, a large family of enveloped, highly diverse, positive-sense, single-stranded RNA viruses, is known to infect humans and animals, resulting in respiratory, hepatic, neuronal and gastrointestinal diseases [3,4,5]. Until recently, HCoVs-NL63, HCoVs-HKU1, HCoVs-229E, HCoVs-OC43, Middle East respiratory syndrome-CoV (MERS-CoV) and severe acute respiratory syndrome-CoV (SARS-CoV) were the six coronaviruses that affected humans [1,5,6,7,8,9,10]. SARS-CoV and MERS-CoV are the coronaviruses responsible for the highest mortality rates in human, 10% and 40%, respectively [11].

Hence, the recently discovered SARS-CoV-2 is the seventh member of the coronavirus family to affect humans. When the genome of SARS-CoV-2 was isolated from patients and analyzed, it was revealed that it is 79.5% similar to SARS-CoV, while being 96% similar to a RdRp region from BatCoV RaTG13, a bat coronavirus [12]. Although believed to originate from bats, it is yet to be determined whether the virus was transmitted directly to humans or through an intermediate host. Minks and Malayan pangolins have been suggested as potential intermediate hosts for the transmission of SARS-CoV-2 from bats to humans [1,13,14].

## 2. Clinical Aspects

Among confirmed cases, it was reported that most (87%) of the patients were aged between 30 and 79 years [15,16]. Almost half the cases have one or several underlying medical conditions, including cardiovascular disease, diabetes and hypertension [17,18,19]. Case studies have revealed that the fatality rates were high among patients with underlying medical conditions [15]. 

**Figure 1:**
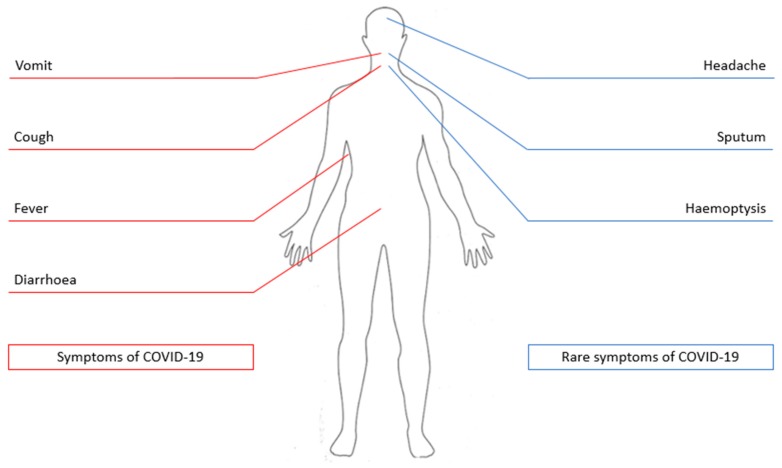
Symptoms of coronavirus disease 2019 (COVID-19). The figure shows the symptoms that may be exhibited by individuals infected with severe acute respiratory syndrome coronavirus 2 (SARS-CoV-2).

The symptoms of COVID-19 include fever (88%), cough (68%), vomiting (5%) and diarrhea (3.7%) as shown in Figure 1 [20]. Less common symptoms include headache, sputum production and hemoptysis [1,15,17,18,19,20,21]. Recently, it has been pointed out that anosmia (loss of smell) and ageusia (loss of taste) may be symptoms due to infection with SARS-CoV-2; however, some patients presented as asymptomatic cases [16].

Most patients had pneumonia and in 70% of patients lymphopenia was observed, while increased prothrombin time as well as an increased level of lactate dehydrogenase were identified in some patients [21]. Radiologic analysis of the lungs revealed ground-glass shadow in the form of patchy ground-glass opacities and patchy consolidation that were mostly located in the middle and outer zone of the lungs [22,23]. Patients with abnormal liver function, with elevated aspartate aminotransferase or alanine aminotransferase, as well as a myocardial zymogram showing elevated levels of lactate dehydrogenase and creatine kinase, were identified [24]. 

COVID-19 cases have been classified as mild, severe and critical types. Mild patients had mild pneumonia while severe patients exhibited dyspnea, increased respiratory frequency and blood oxygen saturation within 24 to 48 h, and critical patients suffered from respiratory failure, acute heart injury, septic shock and multiple organ failure [15,24]. The median incubation period of COVID-19 from first symptoms to dyspnea, admission into a hospital and severe acute respiratory syndrome were 5, 7 and 8 days, respectively [21]. 

Autopsy revealed bilateral diffuse alveolar damage linked with fibromyxoid exudates and accumulation of mononuclear infiltrates, mostly lymphocytes [25]. Microvesicular steatosis and lobular and portal activity were observed in the liver, but it is unclear whether the damage was induced by SARS-CoV-2 or drugs, while interstitial mononuclear inflammatory infiltrates were also found in the heart [25].

## 3. Diagnosis

It has been observed that the main symptom of SARS-CoV-2 infection is fever [20]. Hence, identifying individuals exhibiting a high temperature is an essential step in the diagnosis of COVID-19. Physical examination may help identify patients in a severe condition since they are likely to exhibit shortness of breath, weakened breath sounds, speech tremor and dullness in percussion [15]. Chest X-rays reveal patchy shadows and changes in the lungs, mostly in the outer zone of the lungs, which develop into multiple ground-glass opacity, pulmonary consolidation, infiltrating shadows and infrequent pleural effusion [15]. Computed tomographic (CT) scans of the chest provide clearer images of the pulmonary lesions as compared to X-rays whereby segmental consolidation and ground-glass opacity are observed, mainly in the periphery of the lungs [15].

**Figure 2:**
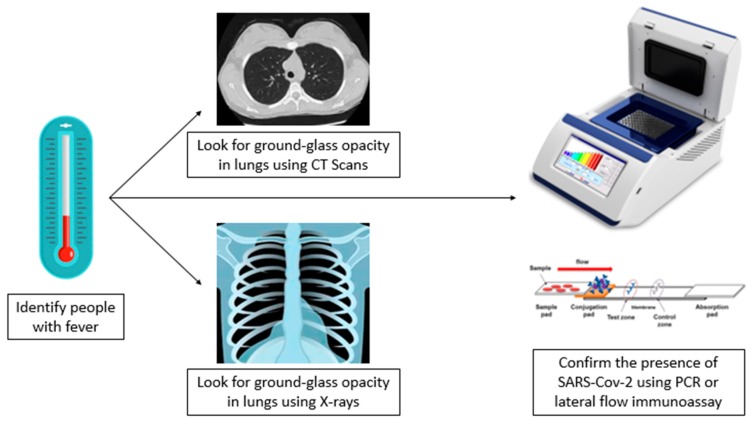
Diagnosis method for SARS-CoV-2. People with fever are identified, then ground-glass opacity is detected using X-rays and CT scans, and the presence of SARS-CoV-2 is confirmed using molecular detection techniques, such as PCR.

Although symptoms and radiographic analysis indicate the possibility of SARS-CoV-2 infection, laboratory diagnosis is definitive since it allows the differentiation of the SARS-CoV-2 infection from other viral infections such as MERS-CoV and SARS-CoV infections [26]. Samples such as swabs, nasal swabs, trachea and nasopharynx extracts, lung tissue, sputum, feces and blood should be isolated and used for testing [26]. The virus can then be detected through polymerase chain reaction (PCR), a molecular diagnosis method that relies on the sequence of nucleic acids for detection [26]. Several primers have been established for the detection of SARS-CoV-2 through real-time reverse-transcription PCR (RT-PCR), which allows for rapid and specific identification of the virus [27]. Immunological methods such as immunofluorescence assay, direct fluorescent antibody assay, protein microarray, semiconductor quantum dots, MAb-based rapid nucleocapsid protein detection and the microneutralization test can be used to rapidly investigate the presence of SARS-CoV-2 [26]. Figure 2 shows the diagnosis method for SARS-CoV-2.

Although the main laboratory technique used to detect SARS-CoV-2 is PCR, other methods have been suggested and developed for the detection of the virus. Clustered regularly interspaced short palindromic repeats (CRISPR) technology has been evoked to detect SARS-CoV-2 by using CRISPR’s ability to hunt down genetic snippets [28]. Immunoassays that use monoclonal antibodies to detect viral antigens or use viral antigens to detect patient antibodies produced against the virus, in the lateral assay format, have been employed for the detection of SARS-CoV-2 [28]. The main advantage of immunoassays, especially in the lateral assay format, is that they can be used to detect the virus in less than 30 minutes without the need for trained personnel or instruments. 

## 4. Mode of Transmission

MERS-CoV was transmitted by close person-to-person contact. Although primary cases of infection have been linked to contact with infected dromedary camels, identified as the reservoir host for MERS-CoV, transmission of the virus occurred via respiratory secretions released by the infected individuals when coughing and sneezing [29]. Similarly, respiratory droplets from coughing or sneezing was the method by which the transmission of SARS-CoV occurred, during person-to-person contact. Although less common, fecal transmission, fomites and handling of animals also resulted in the transmission of the virus [30].

Although not completely clear, the mode of transmission of SARS-CoV-2 is thought to be similar to MERS-CoV and SARS-CoV [29]. Transmission of SARS-CoV-2 is thought to occur from human to human via respiratory secretions released by the infected individuals when coughing and sneezing [19,31]. Asymptomatic carriers may also transmit the virus and hence play a crucial role in the spread of the disease [32]. Recently, the prospect of fecal–oral transmission of SARS-CoV-2 has been raised since the virus has been detected in the feces of COVID-19 patients [33]. This indicates that the virus can exist and replicate in the digestive tract, but it is not clear whether eating SARS-CoV-2-contaminated food causes infection. On another note, perinatal infection with SARS-CoV-2 may cause problems including respiratory distress and abnormal liver function for the newborn, but transmission of the virus through birth is yet to be confirmed as Zhu et al. showed that transmission did not occur [34]. Moreover, it was recently reported that SARS-CoV-2 was able to remain viable in aerosols for more than three hours and that the virus can survive on plastics and stainless steel for up to 72 h [35].

## 5. Epidemiology

**Figure 3:**
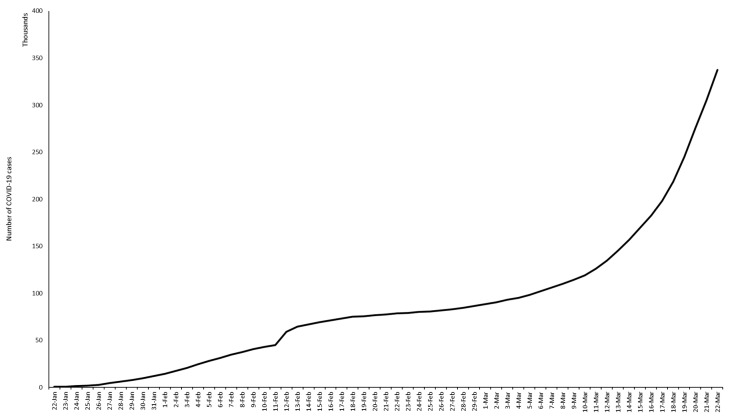
Number of cases of COVID-19. The graph shows the number of cases of COVID-19 reported worldwide over the span of 2 months (22 January 2020 to 22 March 2020) [37].

Our limited knowledge suggests that cases tend to be in groups that occur in waves, developing into larger outbreaks. The first documented outbreak occurred in Wuhan, China [36]. SARS-CoV-2 has infected over 292,142 and killed 12,784 people according to the WHO [2]. Based on the report, 96,580 cases have been confirmed in the Western Pacific region with a death toll of 3502, while 195,511 cases and 10,189 deaths were reported in the European region. In the Southeast Asia region and African region, 1990 and 1305 cases leading to 65 and 26 deaths, respectively, were reported. Some 27,215 cases and 1877 deaths were reported in the Eastern Mediterranean region. The numbers clearly indicate that SARS-CoV-2 is now a worldwide threat. Figure 3 shows the number of cases over the span of two months (22 January 2020 to 22 March 2020). 

The five countries with the most cases of COVID-19 are China, Italy, United States of America, Spain and Germany, as of 24 March 2020 [37]. The total number of cases reported in China is 81,218, of which 73,650 have recovered, 3281 resulted in death, 2888 are in a mild condition and 1399 are in a serious condition [37]. In Italy, out of 69,176 cases, 8326 have recovered, 6820 resulted in death, 50,637 are in a mild condition and 3393 are in a serious condition [37]. The total number of cases reported in the United States of America is 54,968, of which 379 have recovered and 379 resulted in death [37]. With 36,173 mild cases, 2636 serious cases, 5367 recovered cases and 3434 deaths, the total number of reported cases in Spain is 47,610 [37]. As the country with the fifth highest reported number of COVID-19 cases (34,009), Germany has 30,282 mild cases and 23 serious cases, and 3532 patients have recovered while 172 cases resulted in fatality [37]. A total of 8077 cases have been reported in the United Kingdom, of which 7500 are in a mild condition, 20 are in a serious condition, 135 have recovered and 442 have ended in fatality [37].

## 6. Pathogenesis

Virion spike (S) protein, responsible for the receptor binding (S1 domain) and cell fusion (S2 domain) of coronavirus, is essential in determining the host tropism of the virus [38]. S1 and S2 subunits are formed by the cleavage of a precursor, whereby S1 dictates cellular tropism and viral host range with the receptor-binding domain [26]. S2 is divided into heptad repeats 1 (HR1) and 2 (HR2) and mediates fusion of virus with cell membrane when the HR1 attach together and bind to the HR2 to form a six-helix bundle, bringing cell membranes and virus in close proximity to trigger fusion [26]. Using HeLa cells expressing or not expressing angiotensin-converting enzyme II (ACE2) proteins from humans, used as the cell receptor for SARS-CoV entry, it was revealed that SARS-CoV-2 was able to gain entry only in the cells expressing ACE2, suggesting that ACE2 is a cell receptor used by the virus [12]. Structural and biophysical analysis showed that SARS-CoV-2 has 10 to 20 times higher affinity, as compared to SARS-CoV, towards ACE2 [39].

## 7. Treatment

As SARS-CoV-2 is a recently discovered virus, effective treatment options have not been established, hence antiviral drugs, including ribavirin, oseltamivir, ganciclovir, ritonavir and lopinavir, have been used to reduce the development of respiratory complications by reducing viral load [24]. Secondary infections were also observed, and treatments were administered based on the sensitivity of the bacterial cultures to drugs [18]. Some of the general strategies to fight SARS-CoV-2 infection are respiratory support, organ function support, bronchoalveolar lavage, extracorporeal membrane oxygenation and blood purification [15].

## 8. Developing a Vaccine

Spike (S) protein, a major vaccine target for coronaviruses, has been assessed in different types of vaccines designed for infections caused by coronaviruses, including inactivated whole-virus particle, S protein subunit vaccine, virus-like particle with S protein incorporated into influenza virus or hepatitis virus protein, live attenuated virus with gene deletion, virus vectors (such as modified adenovirus carrying S protein) and deoxyribonucleic acid (DNA) vaccine, which encodes part or the full length of the S protein gene [26]. A double inactivated vaccine was developed for SARS-CoV by inactivating the virus using sequential formaldehyde and ultraviolet inactivation, and evaluated in mice models where it was able to elicit the production of high levels of antibodies against the virus [40]. A recombinant SARS virus-like particle vaccine, consisting of influenza M1 protein and SARS-CoV S protein, was developed using the baculovirus–insect cell expression system and tested in mouse models, resulting in reduction of the virus in the lungs to below detectable levels and complete protection from death as well as triggering the production of high levels of antibodies against SARS-CoV [41]. Recombinant SARS-CoV lacking envelope protein was tested in mice and it was shown that the vaccine was able to completely prevent death and resulted in more rapid virus clearance [42]. A small fragment of the S protein of SARS-CoV linked to a human IgG1 Fc fragment was able to induce high titers of antibody production in mice that neutralized infection caused by SARS-CoV [43]. A DNA vaccine encoding the first 725 amino acids of MERS-CoV S protein was shown to induce high titers of neutralizing antibodies in mice, and a DNA vaccine encoding S protein of SARS-CoV was shown to induce the production of neutralizing antibodies in humans in a phase 1 clinical trial [44,45]. The aforementioned methods led to the development of vaccines against SARS-CoV and MERS-CoV that have already undergone phase 1 clinical trials [45,46]. As most coronaviruses have a similar infection pathway and structure, the research strategies mentioned above could be used as the framework for developing effective vaccines against SARS-CoV-2.

## 9. Developing Drugs

Remdesivir is a drug that has recently been shown to possess activity against MERS-CoV [47]. An effective result was reported when remdesivir was used in the treatment of a patient with COVID-19 [33]. This would suggest that strategies similar to those used in the development of treatments against other coronavirus infections, such as SARS-CoV and MERS-CoV, could be used in the development of effective treatments against SARS-CoV-2. 

An anti-SARS-CoV peptide, derived from the HR2 domain of SARS-CoV S protein S2 subunit, that can bind to its HR1 domain, resulting in the inhibition of the S protein-mediated membrane fusion and hence SARS-CoV infection, has been identified [48]. The same technique was used to successfully develop an anti-MERS-CoV peptide that could inhibit infection by the virus [49]. Therefore, this technique could be used to develop an anti-SARS-CoV-2 peptide that could inhibit infection by the virus.

Neutralizing antibodies could be used to target SARS-CoV-2. Previously, mice were immunized with the receptor-binding domain from the S protein of coronaviruses and the antibodies produced by the mice were humanized and isolated [26]. Another approach is the use of phage-display antibody library in combination with cloning of single B cells from human survivors to produce human antibodies [26]. Several neutralizing antibodies have been identified for use against SARS-CoV and MERS-CoV [50,51]. Hence, novel neutralizing monoclonal antibodies could be developed for use against SARS-CoV-2 using the same technique.

Drug repurposing can lead to new treatment options faster than discovery of novel drugs since safety profiles of the available drugs are already available. One method for effectively selecting drugs for repurposing purposes is the use of bioinformatics to analyze the interactions of drugs with the proteins of the target organism, in this case SARS-CoV-2. Bioinformatics was recently used to identify drugs that could target SARS-CoV-2 and 78 drugs were identified as possible repurposed drugs [52]. After refining the list of drugs by removing drugs found unfit for repurposing, based on the effects of the drugs on the symptoms of SARS-CoV-2, and their side effects, 30 drugs were identified [52]. Pseudoephedrine, andrographolide, atiprimod, YSIL6, tapinarof, etanercept, adalimumab, infliximab, chloroquine, epinephrine, thalidomide, clenbuterol, pranlukast, afelimomab, golimumab, siltuximab, olsalazine, ibalizumab, cefazolin, abacavir, myricetin, N-formylmethionine, ruplizumab, framycetin, ketoprofen, maraviroc, vicriviroc, proline, quercetin and artenimol are the 30 drugs that were identified as possible drugs to repurpose for use against SARS-CoV-2. Another study also used bioinformatics tools to identify existing drugs that could be repurposed for use against SARS-CoV-2. Fifteen approved drugs, 4 drugs currently undergoing phase 1 clinical trials and 18 drugs in the pre-clinical stage were identified through chemoinformatics, while 12 approved drugs, 10 drugs currently undergoing phase 1 clinical trials and 10 drugs in the pre-clinical stage were revealed through specialist knowledge [53].

WHO announced that clinical trials involving remdesivir, lopinavir, ritonavir and chloroquine would be conducted, since the drugs showed anti-SARS-CoV-2 activity either in vitro and/or animal studies [54]. However, it was reported that the use of ritonavir and lopinavir in combination did not improve survival or speed recovery [54]. However, WHO announced that the drugs should be tested on a larger scale with a variety of patients [55]. Remdesivir was initially developed to fight Ebola, chloroquine is an anti-malarial drug, while ritonavir and lopinavir are used in the treatment of human immunodeficiency virus [55].

Vitamin C has also been evoked for use in cases of SARS-CoV-2 infection, since at high doses it could result in the immunosuppression of hyperactivation immune effector cells that cause lung injury in COVID-19 cases, while being a safe treatment option [56].

## 10. Challenges Faced due to SARS-CoV-2

SARS-CoV-2 is transmitted easily and is hard to control. As such, there is a shortage in stocks of face masks worldwide, which facilitates transmission of the virus. Due to the overwhelming number of cases and ease of transmission, many countries have issued movement bans. Hence, air travel has been banned in many countries, while countries that still have air travel usually impose 14 days of quarantine for travelers, to identify if they are infected with SARS-CoV-2. One way in which people are being diagnosed is whether they have fever, which is the predominant symptom of SARS-CoV-2 infection. However, many people are lying about showing symptoms, such as fever, and are avoiding temperature checkpoints by taking drugs that suppress fever.

One of the main challenges with COVID-19 are the people that show mild to no symptoms and hence might not be detected as infected [57]. A study has shown that more than 59% of the cases of COVID-19 in Wuhan were not confirmed, asserting the presence of potential asymptomatic and mild-symptomatic cases that may be contagious [58]. After the observation of 13 individuals that tested positive for SARS-COV-2 by laboratory testing for 30 days, it was reported that four of them did not show any symptoms, suggesting the presence of asymptomatic carriers [59]. However, it does not mean that 31% (=4/13) of all COVID-19 cases are asymptomatic. We need to take into account the limitations of statistics due to the sensitivity and detection window (the period during which viral shedding occurs to a detectable level). In support, it has been reported that viral shedding still occurs after symptoms end [60]. The viral loads in both asymptomatic and symptomatic patients were reported to be similar, suggesting a similar transmission potential for people showing mild to no symptoms as compared to symptomatic individuals [61]. The fact that people showing mild to no symptoms may transmit the virus while being hard to detect as carriers, increases the chance of transmission.

## 11. Future Studies 

Future studies should focus on studying the pathogenesis of SARS-CoV-2. By identifying the mechanism by which the virus causes pathogenicity, more insight might be obtained into the various molecules that can be affected by drugs and vaccines to effectively treat and prevent COVID-19 cases. As mentioned above, several methodologies have been used for the development of vaccines against other coronaviruses, such as SARS-CoV and MERS-CoV, and can be explored for the development of vaccines against SARS-CoV-2. Furthermore, considering that SARS-CoV-2 affects the respiratory tract, drug delivery options that effectively deliver drugs into the respiratory tract should be developed. Repurposing drugs that have been identified as potential drugs against SARS-CoV-2 should be screened in vitro to confirm that they have activity against SARS-CoV-2. The drugs that show effects in vitro should then be investigated in animal models, followed by clinical trials. 

## 12. Conclusions

Our current knowledge about SARS-CoV-2 is limited and there is no current treatment or vaccine for COVID-19 cases. The symptoms of COVID-19 include fever (88%), cough (68%), vomiting (5%) and diarrhoea (3.7%), and transmission of SARS-CoV-2 is thought to occur from human to human via respiratory secretions released by the infected individuals when coughing and sneezing. Therefore, preventive measures should be used such as encouraging people to stay at home, discouraging mass gatherings and exercising social distancing to prevent the spread of the virus. Future research should focus on understanding the pathogenicity of the virus, since knowledge about pathogenicity might help in the development of vaccines and effective drugs.
